# An educational intervention to facilitate appropriate subspecialty referrals: a study assessing resident communication skills

**DOI:** 10.1186/s12909-022-03592-4

**Published:** 2022-07-09

**Authors:** Elise A. Stave, Larrie Greenberg, Ellen Hamburger, Mary Ottolini, Dewesh Agrawal, Karen Lewis, John R. Barber, James E. Bost, Ashraf S. Harahsheh

**Affiliations:** 1 Pediatrics, Children’s National Hospital, Washington, DC, USA; 2 George Washington University School of Medicine and Health Sciences, Washington, DC, USA; 3 Maine Medical Center, Portland, ME USA; 4 Division of Cardiology, Children’s National Hospital, 111 Michigan Ave, Washington, DC, NW 20010 USA

**Keywords:** Pediatric residents, Communication, Subspecialty referral, Educational intervention, Syncope, Pediatric cardiology

## Abstract

**Background:**

Our goal was to improve pediatric residents' advanced communication skills in the setting of referral to address the entrustable professional activity of subspecialty referral identified by the American Board of Pediatrics. To accomplish this aim, we created a referral and consultation curriculum to teach and assess core communication skills in subspecialty referral involving an adolescent with syncope, an anxiety-provoking symptom that is rarely associated with serious pathology.

**Methods:**

We utilized blended multimodal educational interventions to improve resident communication skills in referral of patients. Trainees participated in 1) an interactive online module on syncope focusing on “red-flag” symptoms that would warrant a subspecialty cardiology referral and 2) a 4-h intervention with Standardized Parents (SPs), focusing on the case-based application of communication skills. Communication skills were assessed by two pre- and post- Objective Structured Clinical Examination encounters of patients with syncope, with an SP evaluation using a 20-item checklist. Analysis was performed with Sign test and McNemar’s test. Trainees provided feedback on a Critical Incident Questionnaire, which was analyzed qualitatively.

**Results:**

Sixty-four residents participated. There was an overall improvement in communication skills based on SP scores (82.7 ± 10.9% to 91.7 ± 5.0%, *p* < 0.001), and 13/20 items demonstrated significant improvement post-intervention. Residents’ improved performance enabled them to address patient/family emotions, explain referral logistics, and clarify concerns to agree on a plan.

**Conclusions:**

By participating in this curriculum, residents’ communication skills improved immediately post-intervention. Further research is needed to assess if this intervention improves patient care by providing residents with enduring skills to judiciously manage the referral process.

**Supplementary Information:**

The online version contains supplementary material available at 10.1186/s12909-022-03592-4.

## Introduction

Focused subspecialty referral and appropriate consultation are critical skills in providing high-quality care to patients requiring further expertise for addressing potentially serious concerns [1, 2]. Clinicians must recognize red-flag symptoms requiring consultation and subspecialty referral. As importantly, they must demonstrate listening and communication skills to identify the underlying concerns around these issues to address these fears and reassure when appropriate. Primary care pediatricians are often the first to assess children presenting with anxiety-provoking complaints such as syncope. Most children with syncope have no red-flag symptoms meriting diagnostic evaluation or referral, and only 2% have an underlying cardiac etiology [3, 4]. Substantiating this low yield of further cardiac evaluation, 60% of children who were referred to Cardiology for benign simple vasovagal syncope had no medical red-flag criteria for referral [5]. Inappropriate referral can result in unnecessary testing, unjustified use of available resources, and unwarranted financial costs, some of which can be attributed to parental or patient anxiety or demand [6, 7]. Thus, residency training curricula on advanced communication skills are needed to enhance future pediatricians’ skills to manage subspecialty referrals optimally and appropriately.

The American Board of Pediatrics (ABP) recognize referral of patients who require subspecialty consultation as an entrustable professional activity (EPA) and an essential component of a pediatric residency curriculum [8]. To improve pediatric resident confidence and performance in syncope referral, the Children’s National Hospital (CNH) Syncope Education Project was created [9]. During the pilot project, participating pediatric residents on their cardiology rotation completed a 90-min mid-rotation educational workshop focusing on red-flag criteria for cardiology referral and practiced with standardized parents (SPs). Residents exhibited significant improvements in self-efficacy as well as SP rating of their communication [9]. In the current study, we expanded the curriculum to focus on advanced communication skills; for example, offering reassurance to overly concerned parents or negotiating with parents who are not convinced of the need for urgent referral or exercise restriction. Setting the expectation for residents to justify the decision for referral or no referral was an intentional design choice in order to assess educational outcomes on a higher level on traditional taxonomies of clinical assessment. Principles behind our educational intervention include Bloom’s Taxonomy, Miller’s Triangle, and Kirkpatrick’s Four Levels of Training Evaluation [10–12].

Our study hypothesis was that a blended, multimodal interactive educational intervention involving an online learning module followed by a workshop utilizing SP encounters will improve pediatric resident subspecialty referral communication skills in counseling a concerned parent. Our aim was to improve residents' advanced communication skills in the setting of referral assessed by SP ratings and create a referral curriculum that is applicable to a broad range of clinical scenarios.

## Methods

### Settings and participants

**Fig. 1:**
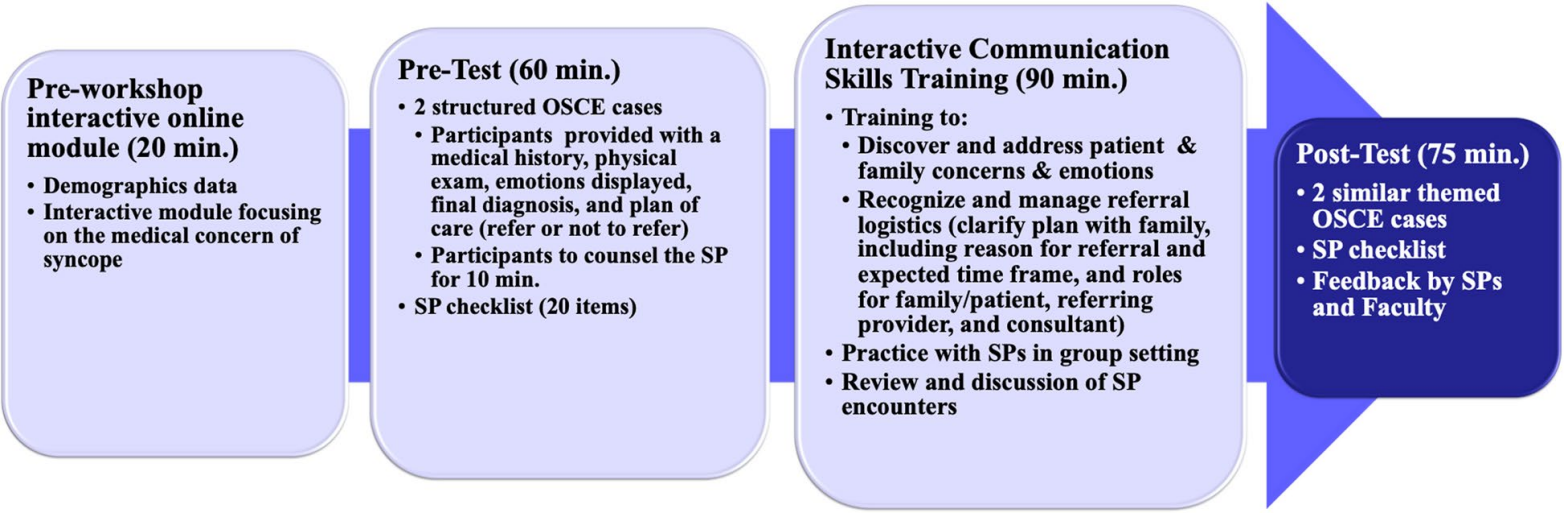
Educational Curriculum. OSCE: Objective Structured Clinical Examination, SP: Standardized parent

We implemented a blended multimodal educational intervention to improve pediatric resident subspecialty referral skills. This intervention included an interactive, online module and an in-person, half-day workshop with SPs (Fig. 1). Residents rotating on electives were recruited to participate during academic years 2015 to 2019, when we conducted the study. Since those residents did not have a primary role in covering patients, no cross coverage for patient care was needed. Participation was voluntary, results were kept private and not shared with the program director, and performance on the activity did not impact standing in the program. The study was conducted at a tertiary pediatric center with 120 pediatric residents, with 40 residents in each of three training years.

### Resources: involvement of standardized parents and faculty

Two SPs were hired for a 4-h timeframe to work with residents as well as for a 2–4 h curriculum-specific training session; over the course of the study, five SPs were utilized in total. Each session could accommodate up to 4 residents, with one half-day session per month. The costs associated with this program included $2250 for consultation, curriculum development support, staff time, and use of the CNH Simulation Center. Additionally, costs of SPs were ~ $25/hour. A faculty member (author ASH) spent 3% FTE each month supervising these sessions and facilitating the workshop.

### Training of standardized parents

SPs were trained by an SP Educator (author KL) from the Clinical Learning & Simulation Skills (CLASS) Center at The George Washington School of Medicine and Health Sciences. This training included learning scenarios that addressed the need for referral. As part of these scripts, authors KL and ASH coached the SPs in how to behave emotionally within the context of each scenario and how to respond to residents’ potential comments and questions. To pilot the study and determine if there were gaps that needed addressing before beginning interactions with residents, a practice session was coordinated with a senior Cardiology fellow simulating a resident to train the SPs and fine-tune their responses. The fellow and SPs practiced and discussed responses until SPs had standardized responses. Training design and implementation followed the Association of Standardized Patient Educators (ASPE) Standards of Best Practice (SOBP) [13]. The SPs at the CLASS Center routinely participate in high stakes sessions with George Washington University medical students in addition to curricula such as ours; SPs routinely roleplay and then provide feedback in each of these scenarios.

### Specifics of educational curriculum

The interactive, online module (which can be viewed at http://www.childrensmedicaleducation.org/review/syncope/) provided education on the medical concern of syncope, particularly about “red-flag” symptoms that would point towards a cardiac etiology of syncope (i.e. exertional syncope, history of sensorineural hearing loss, family history of sudden death, etc.) [5, 14]. The module was created using Articulate Storyline in collaboration with instructional design expertise at the Children’s National Office of Medical Education. Additionally, this module reviewed how to educate patients and families on benign vasovagal syncopal episodes and what steps to take for the future if the patient feels faint, such as counter-pressure exercises [15]. This module required answering questions before progressing, such as indicating what you would ask a patient on initial presentation or matching symptoms with a particular type of syncope. Residents were tasked with completing the 20-min interactive online module the night prior to the in-person communication skills workshop.

For the half-day in-person workshop, trainees participated in two pre-intervention Objective Structured Clinical Examination (OSCE) cases, one requiring subspecialty cardiology referral and the other not requiring referral, as well as two post-intervention OSCEs with the same themes. The OSCE was designed by authors ASH, LG, and KL. Regardless of the need for referral, the SPs related concerns that the trainee was tasked with resolving during the encounter. For example, the SPs requiring referral had reasons why it might be hard to attend the subspecialty appointment or to limit exercise (i.e. their child had an upcoming athletic competition that could result in a college scholarship), requiring a thoughtful response to persuade them of the severity of the presenting problem and urgency for referral. On the other hand, for the case in which referral was unnecessary, the SPs were instructed to ask challenging questions, such as: “I just read an article about sudden death in an athlete—what are the chances my child will be the next one to die on the field?” Using a 20-question checklist, the SPs assessed the residents in all OSCE encounters, with some questions requiring the SPs to rate participants on a Likert scale from strongly disagree to strongly agree and other questions calling for yes/no responses. The SP checklist was modified from a previously validated SP checklist developed using the Set the stage, Elicit information, Give information, Understand the patient's perspective, and End the encounter (SEGUE) framework, which provides a guideline to evaluation for specific communication tasks in a medical encounter with the goal to achieve high inter-rater reliability between SPs [9, 16]. Utilizing the framework, along with comprehensive training with thoughtful fine-tuning, are both required to attain optimal reliability [16]. In creation of the checklist for this study, there was a focus on using “discrete items” and “clear coding rules” in making the checklist for SP evaluation of residents and referral-specific items were in nominal format [9, 16]. Each SP was trained how to use the SP checklist and given time to ask clarifying questions. As trainees did not obtain a history or perform a physical examination, the door chart of the encounter for the trainee included the history, physical exam findings, diagnosis, and whether or not referral was indicated. Trainees were also provided with a list of the emotions displayed by the family on the door chart, such as “fear” and “surprise” for the patient not requiring referral. The trainees were tasked specifically with counseling the SP within the 10 min encounter about the diagnosis and advised treatment plan.

The interactive workshop also included: (1) review of the principles of referral, (2) a refresher on “red-flag” symptoms of syncope, (3) an interactive communication skills intervention on addressing underlying emotions and concerns and on managing referral logistics, and (4) practice with SPs in a group setting (Fig. 1). During practice with SPs in a group setting, residents watched one of their peers practice the challenging components of the referral conversation with an SP in front of the group and received immediate feedback.

### Feedback

After residents completed the pre-test OSCEs, the SPs provided individual and group feedback about performance, specifically remarking on positive behaviors that were observed. The faculty used these comments to make teaching points. After completion of all OSCEs, SPs and faculty provided feedback to any resident interested in further feedback.

Trainees provided feedback on the curriculum by completing the Brookfield Critical Incident Questionnaire at the end of their SP interaction to characterize which factors enhanced or hindered learning [17]. These qualitative comments were categorized, enumerated, and summarized independently by three co-authors (EAS, LG, and DA) to identify key themes. The themes with the highest number of representative comments were included in Table 4.

### Institutional review board approval

The Children’s National Hospital Institutional Review Board approved this study with written informed consent obtained from all participants.

#### Statistical analysis

Statistical tests for paired data were utilized for all analyses. Analysis of the SP checklist results was performed with the Sign test and McNemar’s test, with the Sign test utilized for questions that were graded by SPs on a Likert scale from “strongly agree” to “strongly disagree”. Additionally, Likert scores were converted from 1–5 to 20–100 with 1 corresponding to 20, 2 corresponding to 40, and so on, in order to report data out of 100%. Since they were paired discrete variables with limited variability, the Sign test, a non-parametric alternative to the paired t-test, was utilized. McNemar’s test was used for items graded by SPs on a binary scale (yes/no) to evaluate for pre-post differences. A total percent score was calculated as a composite of the average of all SP items where binary variables were converted to 0/100. This total score was analyzed by paired t-test as it was normally distributed. A Cronbach alpha score was also calculated for the SP checklist to assess reliability. Demographics were analyzed by simple linear regression analysis to determine if any were associated with the difference between pre- and post-intervention total scores.

## Results

Sixty-four trainees volunteered to participate in the study; 54 participants completed a demographic questionnaire, of whom 74% were women (Table 1).

**Table 1:** Participant demographics

	**Number**	**Percentage**
**Sex**		
Female	40	74%
Male	14	26%
**Year of training**		
PGY-1	29	54%
PGY-2	7	13%
PGY-3	17	31%
PGY-4	1	2%
**Previous Communication Skills Exposure**		
Communication Skills workshop in medical school	29	54%
Communication Skills workshop in residency	13	24%
Communication Skills workshop/curriculum in medical school that covered the “Difficult Patient Encounter”	34	63%
Communication Skills workshop/curriculum in residency that covered the “Difficult Patient Encounter”	8	15%
**Exposure to patients with presenting symptom of syncope**		
None	3	5%
1–3 patients	23	43%
4–6 patients	15	28%
> 7 patients	13	24%

*PGY* Post-graduate year

Significant improvement was seen in the SPs’ rating of resident communication skills pre-intervention compared to post-intervention (82.7 ± 10.9% versus 91.7 ± 5.0%, *p* < 0.001) (Table 2). A Cronbach alpha score was calculated to evaluate checklist reliability (Cronbach alpha = 0.71). Significant improvement was noted in 13 of 20 items on the SP checklist, including but not limited to the following checklist items: “The resident asked me what was the most concerning factor for us today”, and “The resident noted that I seemed sad/mad/distressed/worried”. The trainees improved in 4 of 7 referral-specific metrics, including: “the resident explained what you could expect when you see the Cardiologist,” which increased from 51.9% to 75% post-intervention (*p* = 0.01). For OSCEs aimed to reassure the family that referral was not indicated, a significant improvement was noted for this checklist item: “The resident successfully helped you to feel reassured that this is not a life-threatening condition” (pre-post scores of 82.6% and 97.8%, respectively, *p* = 0.008).

**Table 2:** Pre- and post-intervention results for standardized parent Objective Structured Clinical Examination (OSCE) by checklist item

	**Pre-intervention (*n* = 64)**	**Post-intervention (*n* = 63)**	
	**Mean (SD)**	**Mean (SD)**	*P*-value*
1. The resident showed interest in me as a person	83.9 (11.1)	91.1 (10.8)	** < 0.001**
2. The resident made me feel that he/she was glad that I brought my child in today	81.4 (11.9)	88.3 (10.4)	** < 0.001**
3. The resident used words that I understood	83.4 (11.2)	89.5 (9.2)	**0.003**
4. The resident used nonverbal behaviors that conveyed attentive listening	83.6 (11.6)	91.3 (9.4)	** < 0.001**
5. The resident asked me what was the most concerning factor for us today	55.5 (40.9)	82.5 (27.2)	** < 0.001**
6. The resident asked me how I feel about the situation	64.8 (40.5)	78.6 (30.7)	0.071
7. The resident validated my concern/feelings	97.7 (13.9)	97.6 (10.7)	1.000
8. The resident noted that I seemed sad/mad/distressed/worried	58.6 (40.4)	73.0 (34.6)	**0.024**
9. The resident made empathetic statements (That must be difficult)	93.8 (18.9)	93.7 (21.0)	1.000
10. The resident engaged me in an exchange to arrive to the plan	87.5 (28.2)	97.6 (10.7)	**0.012**
11. The resident made a final decision with regard to referral	98.4 (8.8)	98.4 (8.8)	1.000
	**% (n)**	**% (n)**	
12. If no referral was made, the resident successfully helped you to feel reassured that this is not a life threatening condition	82.6 (38)	97.8 (45)	**0.008**
13. If a referral was made, the resident helped you understand the reason for the referral	96.1 (49)	98.0 (50)	0.564
14. If a referral was made, the resident explained that this could be a life threatening condition	70.6 (36)	80.4 (41)	0.166
15. If a referral was made, the resident explained what you could expect when you see the Cardiologist	51.9 (27)	75.0 (39)	**0.011**
16. If a referral was made, the resident recognized and managed the logistics of the referral	76.9 (40)	96.2 (50)	**0.002**
17. If a referral was made, the resident clarified plan with the family and ensured conceptual understanding and agreement on logistics of the plan	76.0 (38)	98.0 (49)	**0.002**
18. If a referral was made, the resident explained that exercise restriction is needed	84.3 (43)	100 (51)	**0.005**
19. If a referral was made, the resident decided on the urgency of the referral	90.4 (47)	98.1 (51)	0.103
	**Mean (SD)**	**Mean (SD)**	
20. Provided ongoing patient care and informed us when to call her/him back	60.3 (38.3)	80.2 (35.4)	** < 0.001**
**Total Score**	82.7 (10.9)	91.7 (5.0)	** < 0.001**

^*^Analysis by Sign test for items 1–11, 20 (Likert distribution of data); McNemar’s test for items 12–19 (Nominal data); Paired t-test for total score

*SD* Standard deviation

Analysis of demographic variables to assess for possible associations with difference in score pre- and post-intervention showed no association of year of workshop, sex, age, level of training, prior communication workshop in residency or medical school, or experience with patients presenting with syncope (Table 3).

**Table 3:** Associations of demographic variables with difference between pre- and post-intervention total scores

	**Beta**	**Standard Error**	***P*-value**
Year of workshop	0.540	1.137	0.637
Sex	2.817	3.585	0.436
Age	-0.028	0.668	0.966
Level of Training	0.320	1.638	0.846
Prior Communication Skills workshop in Residency	3.078	3.581	0.394
Prior Communication Skills workshop in Medical School	4.501	3.029	0.143
Prior exposure to patients with presenting symptom of syncope	0.833	1.719	0.630

Qualitative review of the feedback on the Critical Incident Questionnaire revealed six themes of factors that improved learning, with the three highest including: 1. Timely feedback on performance; 2. Group session observing peers interacting with SPs; and 3. Case discussion (Table 4). Additional areas of value included interactively working with SPs as well as specific teaching on communication skills, including general principles and skills specific to referral. As to activities trainees cited as hindering learning, the main themes were the structure of the session (repetition of cases, filling out forms, and downtime) and learner passivity.

**Table 4:** Qualitative analysis of feedback on critical incident questionnaire

Category	Number of comments	Representative quotations
***Factors that improved learning***		
Feedback on performance	32	• “While doing the intervention—it was helpful to engage in the encounter and then get real time feedback on tips to use while speaking with parents. They were useful, practical tips.” • “I was most engaged while getting active feedback during the practice scenarios. I felt this was helpful to point ways to improve in connecting with the family to both address their concerns and make sure they understand my recommendations.”
Group session observing peers interacting with SPs	24	• “The practice SP intervention sessions were extremely valuable, especially with real time feedback from the SPs regarding communication skills and specific key words.” • “I was most engaged while other [participants] were presenting in front of the group and then we discussed what they did well and other suggestions for connecting better with the patient and their family in the future.” • “The active discussion of standardized patient encounters that occurred during the teaching time—we were able to pause, re-direct the encounters and then re-try after discussion, which I found very helpful.”
Case discussion	15	• “When we were going through scenarios as a group and brainstorming better ways to address common issues that come up.” • “I felt most engaged during the facilitated debrief of the standardized patient sessions.”
Interactively working with SPs	12	• “Definitely during the counseling of the SPs, putting theory into practice.” • “I felt engaged during each case encounter.”
Communication Skills: General Principles	10	• “Discussion of techniques in communicating challenging information. Focus on specific language.” • “Specific strategies for approaching patients and family who were more difficult to convince of the utility of going through a particular therapeutic plan.”
Communication Skills: Specific to Referral	7	• “Discussion of when and how to refer patients, specifics of dealing with difficult patients who desire a referral in spite of lack of indication” • “Specific information to give a patient when referring them to a specialist”
***Factors that hindered learning***		
Structure of session	15	• “The post-encounter sessions where we had to then go in to two additional sessions with standardized patients. I felt that it was repetitive. To better assess what was learned, I would have preferred a time-delayed post-encounter perhaps a few days after review of the intervention.” • “It was really frustrating to have so much wasted down time during the morning.” • “Filling out forms”
Learner passivity	8	• “When we were going through the PowerPoint, especially since it was a little more difficult to see.” • “The lecture conveyed very interesting information, but the presentation portions of lectures are always the hardest to pay attention to.”

*SP* Standardized parent

## Discussion

Participation in this blended, multimodal interactive curriculum improved the immediate, post-intervention communication skills of residents in the setting of subspecialty referral. Residents showed a measurable, significant immediate improvement in most communication areas, including the majority of those focused on recognizing and addressing parent emotions and concerns as well as sharing and managing referral logistics. Of the Kirkpatrick levels of education, this curriculum focuses on level 2C, knowledge, specifically skills acquisition [12, 18]. Additionally, our curriculum meets the level of “evaluate” per Bloom’s Taxonomy and “Shows” in Miller’s Triangle [10, 11]. Residents improved in recognizing and managing logistics of referral, clarifying the plan with the family, ensuring conceptual understanding and agreement on logistics. The authors suggest that the curriculum we developed for our study is generalizable to other clinical scenarios requiring referral and justify broadening this intervention to include other patient scenarios.

It is important that residents were able to identify parental anxiety about an emotionally charged diagnosis and develop competence in communicating with a simulated parent about this issue. Forrest et al. reported 16.7% of pediatric subspecialty referrals were influenced by parental or patient anxiety; moreover, Harahsheh et al. found 14% of low yield echocardiograms were obtained for the same reason [6, 7]. A trusting relationship between the physician and in this case parents is paramount to understanding the need to refer or not and can be conveyed in an evidence-based manner as in our study. Whereas the focus of this study was around the diagnosis of syncope with the goal to improve resource utilization and decrease inappropriate referrals, there are many diagnoses in pediatrics and other specialties in medicine that are similarly concerning to patients and their families in which the curriculum we describe could be utilized to train practitioners.

The strengths of this intervention include demonstration of successful application of learned communication skills, utilization of SPs, and the ability for trainees to participate during elective time. Tasked with counseling SPs about whether or not their child required subspecialty referral, residents were highly successful in explaining this challenging, high stakes topic to families. These valuable skills may be generalizable to other potentially serious medical concerns. Trainee responses indicated what they learned from the experience; for example, one participant shared, “I was most engaged while getting active feedback during the practice scenarios. I felt this was helpful to point ways to improve in connecting with the family to both address their concerns and make sure they understand my recommendations”. Participants also valued review of specific language they could use with families, especially for “approaching patients and family who were more difficult to convince of the utility of going through a particular therapeutic plan”.

Another strength of this intervention is that no association was found between SP scores and the year of the workshop, suggesting consistency of our methodology and results over time. An interesting finding was that participants’ prior communication skills workshop experiences and exposure to patients with the presenting symptom of syncope were not associated with improvement in performance. Trainees at all levels can benefit from this intervention and residency programs may benefit from implementing similar curricula to improve residents learning surrounding subspecialty referrals, a skill endorsed by the ABP.

These results build on our prior work with pediatric trainees in improving skills of subspecialty referral, particularly in providing advanced communication tools to counsel a concerned or anxious parent [9]. The improvement in performance in our intervention parallels the improvement reported by Guse et al., in which diagnostic work-up decreased in patients with low-risk pediatric syncope in the pediatric emergency department after a quality improvement intervention introduced a new syncope guideline to providers [3].

The next steps for the project include refining the curriculum based on resident feedback and adding a component to assess for long-term gains by the residents or communities in which they practice, rather than focusing on the short-term achievements. To decrease resource utilization and address resident feedback about the intervention seeming “repetitive” by the end of the day, the curriculum could be changed to eliminate the pre/post OSCE portions. The residents will still be required to do the online portion of the project a day earlier and emerge into the interventional workshop on the day of the curriculum. To evaluate residents long-term, we propose assessing residents performance with real patients in their continuity clinics, perhaps through a quality improvement project similar to prior work showing a decrease in low-probability chest pain referrals to cardiologists, which would assess residents at Kirkpatrick level 4 (results) [1, 18]. Lastly, one could assess change in residents performance by querying their clinic preceptors or subspecialists receiving their referrals for appropriateness of referrals, also evaluating Kirkpatrick level 4 [18].

Residency programs have found innovative ways to improve skills of subspecialty referral; for example, the University of Chicago Internal Medicine Department created a dedicated training program for interns to improve their comfort and skills in requesting subspecialty referrals within the hospital [19]. Additionally, after a self-assessment by residents indicating that they would value further training in subspecialty referral, a team at Children’s National and George Washington University created tools to do so in the primary care and subspecialty arenas, respectively: a referral feedback form and a subspecialty learning prompt [20]. These tools led to resident reflection, during which 57% of residents reported they would change their future practice with the understanding of “new medical knowledge including red flags for referral”, including 17% of residents specifically sharing that they would pursue further management in primary care before referral [20].

SPs have been successfully utilized in education of trainees in various contexts, including assessing Accreditation Council for Graduate Medical Education (ACGME) milestones of residents and medical students. Many studies have also shown short-term improvement in the ability of trainees to share challenging news after working with SPs, as evidenced by a meta-analysis of 17 studies on this topic [21]. Additionally, trainee confidence improves after communication skills interventions with SPs [9, 22–24].

Limitations we identified include: 1) Ten residents did not complete the demographic questionnaire; 2) Study constraints limited our participant numbers; 3) This was a single institution study that may not be generalizable to other training programs; 4) The authors did not study long-term and patient-centered outcome assessments, which logically would follow this report as part of a multi-institutional study, and 5) The potential of smaller residency programs emulating this study might be limited based on resources available. Additionally, the one group pretest–posttest design, while making the study more feasible from a resource standpoint, has disadvantages; to reduce challenges of this design, the study conditions were controlled, participants remained on site for the 4-h time window, and there was a limited time interval between the pretest and posttest [25]. Another limitation includes that the same SPs were utilized in pre- and post-test sessions, playing different patients/families. To counter this potential conflict, the majority of questions SPs were given to evaluate residents were yes/no and all questions were made to be as objective as possible with adaptations of the SEGUE framework and intensive SP training to reduce bias in assessment and optimize inter-rater reliability.

## Conclusion

By participating in this blended, multimodal learning curriculum on the referral and consultation process, residents’ short-term communication skills notably improved. This simulation-based learning is a valuable supplement to resident training to improve specific skills involved in subspecialty referral by enhancing the communication needed for a successful referral despite challenging family circumstances. Further research is needed to assess if this intervention improves the patient experience and patient care by providing residents with enduring skills to judiciously manage the referral process.

## Supplementary Information

**Additional file 1.** All names listed in supplementary material were assigned to Standardized Parents and no names listed represent the names of actors or participants in the study.

## Data Availability

The datasets used and/or analyzed during the current study are available from the corresponding author on reasonable request.
