# Supplementary material for: An educational intervention to facilitate appropriate subspecialty referrals: a study assessing resident communication skills
Source: BMC Med Educ. 2022 Jul 9;22:533. doi: 10.1186/s12909-022-03592-4 (PMC9270829; doi:10.1186/s12909-022-03592-4)
Supplement: Supplementary file 1 — Additional file 1. All names listed in supplementary material were assigned to Standardized Parents and no names listed represent the names of actors or participants in the study. [file 12909_2022_3592_MOESM1_ESM.doc]

**Appendix 1 Standardized Parent Script**

| **Standardized Parent (SP) Script for Pre Test Case 1—Marie & Stephen Davis***  Identifying Case Factors:  Presenting complaint: My son passed out during running a race today.  Length of patient encounter: 10 minutes  Length and type of learner post-encounter (if applicable): 5 mins. SP checklist assessment  Learning objectives/case purpose: Evaluation of a program that teaches residents communication skills in the context of subspecialty referral  Diagnosis: no referral to cardiologist  Learner level: Pediatric Residents  Type of Case:  Teaching  Assessment  Communication  History only  SP Demographics:  Name: Marie Davis (and son Stephen)  Gender: F  Age range: Marie 39-45, Stephen is 17 but he is not present.  Ethnicity: any  Case Information  Setting: pediatrician’s office, 2:00 pm  SP opening statement:  My son passed out during running a race today. The coach said I should bring him to the pediatrician right away.  History of present illness:  What happened: You were watching Stephen at the track meet. Stephen finished running 400 m race and came in 1st place. It was his best time ever. He ran his hardest and it is a hot, humid May day. (The high temperature for the day is 85 degrees F.) He collapsed on the stage after standing on the track for 45 min waiting for the audio system to be fixed for the award ceremony.  Where: The meet took place on the University of Maryland campus.    When: He ran the race at 9:30 am. The incident occurred at 11 am.  Symptoms: He described having blurry vision, feeling weak and then waking up 30 seconds later, according to his classmates. He did not feel any chest pains or palpitations, and none of the observers indicated that there were any jerking episodes. When he woke up, he was a little pale and “a little out of it,” but he came back to normal quickly.  Past medical history:  He has never mentioned being dizzy before and has had no prior problems like this. His pediatrician diagnosed him with a heart murmur as a baby (you were not living in the DC area then), but he grew out of it. Stephen was diagnosed with ADHD he was 8. He usually takes Adderall therapy (20 mg 1 time per day) but since he didn’t have classes today, he did not take it. He has been on Adderall for 8 years and his dose has increased to 60 mg/day. He takes it on weekdays during the school year but not during summers.  Family medical history:  His great grandfather died at 81 years of age, paternal great grandmother had a stroke at 92 years of age. His paternal grandfather has hypertension and his maternal grandmother has diabetes mellitus. There is no history of fainting in the family.  Social history:  Stephen is a high school junior. He runs track and is hoping for a college scholarship. He is also active in the student council. He does well in school and you do not believe he has tried drugs or alcohol. He lives at home with you, and his younger sister, Emily, age 12. You are a single mother (divorced 5 years ago) who has taken the maximum days allowed for the Accrued Sick and Safe Leave Act (ASSLA) sick leave from work to take care of Emily who has Asthma. Your boss has threatened to terminate you if you take any more days off.  Diet:  He eats a reasonable diet. He ate breakfast--juice, banana and cereal at 5:30am. For lunch today, he had a tuna sandwich, chips, and an apple.  Diagnosis:  The learner should say it is simple (neurocardiogenic) fainting. No referral to cardiologist is needed.  How you feel about the situation:  At first you were surprised. It seemed like the coach was overreacting to a simple instance of fainting. Now that you’ve had several hours to think about it, you are starting to get scared. Could this be a serious health condition that could have serious implications for your son’s long term goals?  Most concerning factor:  You just read an article about a basketball player who died suddenly. In addition, a friend of your son's, who is a year older, was recently diagnosed with a serious heart problem that was previously undetected until he collapsed during a track meet in college. You don’t know the details, but could the same thing happen to Stephen? He did have a heart murmur when he was a baby.  Patient presentation:  Remember that this encounter “begins” in the middle. You and the resident have to imagine that the resident has already taken your history. He/she stepped out to answer a page and now is walking back in the room to counsel you. You have a very worried and anxious affect when the doctor walks in. You don’t cry but your body language and tone of voice should demonstrate that you are concerned.  Based on the resident’s plan, you will have one of two anxious responses. (1) If the resident tells you that nothing is seriously wrong with your son, you are not relieved and ask for a cardiology referral. After all, can the resident guarantee he/she is right? Isn’t it better to get a second opinion? (2) If the doctor tells you that Stephen could have a serious condition and needs to see a specialist, you are anxious because it could be serious AND because missing another day from work may get you fired.  The point of the training is to enable the resident to better handle a “difficult” encounter. Your anxiety level should ultimately decrease for those residents who employ some of the following communication strategies:  Elicit emotions via direct and indirect questioning   - Direct: How does this make you feel? - Patient explanatory model: “Do you have any thoughts on what’s behind this?   Offer support and empathy.   - Name the affect: (You are sad/mad, etc.) - Validate (Your child had fainted and you are worried something bad will happen; that’s a lot for any parent to take” - Align (I want to do everything in my power to help you feel assured)   Use nonverbal behaviors that convey attentive listening   - Thoughtful nodding - Occasional Silence - Few hand gestures - Adequate facial expression - Leaning forward   If the doctor does not attempt any of these strategies, (1) for no referral, end your push for the specialist consultation with: “I will think it over tonight, but I can’t guarantee that I won’t seek a specialist tomorrow.” (2) If the doctor recommends you see a specialist, reluctantly agree to whatever strategy the resident comes up with for dealing with your work situation.  Additional Materials:  SP Checklist/scoring rubric  **Presenting situation/door chart information**  Patient Name: Marie Davis and 17-year-old son, Stephen. This is an established patient of yours who was seen for a reassuring school physical 3 months ago. He was noted to have an innocent heart murmur in the past.  Location: Pediatrician’s office  Time/Date: 2:00 pm, middle of May  Hx: Stephen passed out today 45 minutes after track meet (no SOB, chest pain or palpitation) with reassuring PMH (no history of cardiac or seizures), family history (no SIDS, unexplained death, drowning or car accidents, pacemakers) and normal examination. He has benign vasovagal syncope. Mother is anxious.  Medical Information: Stephen’s physical examination is normal  Task: You have already met with the family, obtained the history portion and performed the physical examination. During that time the mother’s emotions displayed were fear and surprise. You had to step outside to answer a page and now you will counsel the family.  Please counsel Stephen’s mother, Marie, as appropriate.  Time: 10 minutes  **SP Script for Intervention Case 1—Laura & Amber Holt***  Identifying Case Factors:  Presenting complaint: My daughter passed out during a swim meet today.  Length of patient encounter: 10 minutes  Length and type of learner post-encounter (if applicable): 5 mins SP checklist assessment  Learning objectives/case purpose: Evaluation of a program that teaches residents to assess syncope in adolescents  Diagnosis: no referral to cardiologist  Learner level: Pediatric Residents  Type of Case:  Teaching  Assessment  Communication  History only  SP Demographics:  Name: Laura Holt (and daughter Amber)  Gender: F  Age range: Laura 39-45, Amber is 15 but she is not present.  Ethnicity: any  Case Information  Setting: pediatrician’s office, 2:00 PM  SP opening statement:  My daughter fainted during a swim meet today. The coach said I should bring her to the pediatrician right away.  History of present illness:  What happened: You were not at the meet. The coach called you right after it happened. Amber finished a high school swim meet and came in 1st place. (She swims the 100 meter freestyle and 100 meter backstroke.) She just finished getting dressed after a long hot shower. She mentioned swimming extra hard because her new boyfriend was watching her. She collapsed in the locker room.  Where: The meet took place on the high school campus.    When: She swam at 9:30 am. The incident occurred at 10:30 am.  Symptoms: There was no dizziness, palpitations nor chest pain. She described having blurry vision, feeling weak and then waking up 30 seconds later, as reported by her teammates. There were no jerking episodes noted. When she woke up, she was “a little groggy and pale, but it passed quickly.”  Past medical history:  She has never fainted before as far as you know and her past medical history revealed no prior problems like this. She had sinus arrhythmia noted on a pre-sport evaluation, but the doctor said it was nothing to worry about. She had what seemed like an upper respiratory infection 2 weeks ago. You gave her over-the-counter medications and it went away after several days.  Family medical history:  You and her father have high blood pressure that you take medication for (enalapril). Everyone else in the family is fine.  Social history:  Amber is a high school sophomore. She is part of the swimming team and is enrolled in advanced classes and wants to go to Johns Hopkins on an academic scholarship. She has never tried cigarettes, drugs or alcohol as far as you know. She lives at home with you, her father and her younger brother, Allen, age 13. Your husband injured his back at work and is at home on disability. He needs a lot of of assistance, cannot drive, and has to go to PT regularly. You have taken the maximum days allowed for ASSLA (sick leave from work) to take care of your husband. Your boss has threatened to terminate you if you take any more days off.  Diet: She has a normal active teenager diet. Today for breakfast she had juice, banana and cereal at 5:30am. She had a Subway turkey sandwich for lunch on the way to the doctor’s office.  Physical examination findings:  The learner will have this information: Amber’s physical examination is normal.  Diagnosis:  The learner should say it is simple (neurocardiogenic) fainting. No referral to cardiologist.  How you feel about the situation:  At first you were surprised. It seemed like the coach was overreacting to a simple instance of fainting. Now that you’ve had several hours to think about it, you are starting to get scared. Could this be a serious health condition that could have serious implications for your daughter’s long term goals?  Most concerning factor:  Your stepbrother (therefore, not a blood relation) was recently diagnosed with a serious heart problem that required a pacemaker. He is younger than you, is very athletic, had no health issues, and just collapsed one day. You don’t know the details but you are very anxious and upset that Amber could have a similar problem. She had what seemed like an upper respiratory infection 2 weeks ago. Could that have been a sign that something serious was wrong?  Patient presentation:  Remember that this encounter “begins” in the middle. You and the resident have to imagine that the resident has already taken your history. He/she stepped out to answer a page and now is walking back in the room to counsel you. You have a very worried and anxious affect when the doctor walks in. You don’t cry but your body language and tone of voice should demonstrate that you are concerned.  Based on the resident’s plan, you will have one of two anxious responses. (1) If the resident tells you that nothing is seriously wrong with your son, you are not relieved and ask for a cardiology referral. After all, can the resident guarantee he/she is right? Isn’t it better to get a second opinion? (2) If the doctor tells you that Stephen could have a serious condition and needs to see a specialist, you are anxious because it could be serious AND because missing another day from work may get you fired.  The point of the training is to enable the resident to better handle a “difficult” encounter. Your anxiety level should ultimately decrease for those residents who employ some of the following communication strategies:  Elicit emotions via direct and indirect questioning   - Direct: How does this make you feel? - Patient explanatory model: “Do you have any thoughts on what’s behind this?   Offer support and empathy.   - Name the affect: (You are sad/mad, etc.) - Validate (Your child had fainted and you are worried something bad will happen; that’s a lot for any parent to take” - Align (I want to do everything in my power to help you feel assured)   Use nonverbal behaviors that convey attentive listening   - Thoughtful nodding - Occasional Silence - Few hand gestures - Adequate facial expression - Leaning forward   If the doctor does not attempt any of these strategies, (1) for no referral, end your push for the specialist consultation with: “I will think it over tonight, but I can’t guarantee that I won’t seek a specialist tomorrow.” (2) If the doctor recommends you see a specialist, reluctantly agree to whatever strategy the resident comes up with for dealing with your work situation.  Additional Materials:  SP Checklist/scoring rubric  **Presenting situation/door chart information**  Patient Name: Laura Holt and daughter Amber, age 15. This is an established patient of yours. She had sinus arrhythmia noted on a pre-sport evaluation, but it was nothing to worry about.  Location: Pediatrician’s office  Time/Date: 2:00 pm  Hx: Amber fainted today an hour after a swim meet (no SOB, chest pain or palpitation) with reassuring PMH (no history of cardiac or seizures), family history (no SIDS, unexplained death, drowning or car accidents, pacemakers) and normal examination. She has benign vasovagal syncope. Mother is anxious.  Medical Information: Amber’s physical examination is normal  Task: You have already met with the family, obtained the history portion and performed the physical examination. During that time the mother’s emotions displayed were fear and surprise. You had to step outside to answer a page and now you will counsel the family.  Please counsel Amber’s mother, Laura, as appropriate.  Time: 10 minutes  **SP Script for post test Case 1—Shelly and Jen Turner***  Identifying Case Factors:  Presenting complaint: My daughter passed out during a lacrosse game today.  Length of patient encounter: 10 minutes  Length and type of learner post-encounter (if applicable): 5 mins SP checklist assessment  Learning objectives/case purpose: Evaluation of a program that teaches residents to assess syncope in adolescents  Diagnosis: no referral to cardiologist  Learner level: Pediatric Residents  Type of Case:  Teaching  Assessment  Communication  History only  SP Demographics:  Name: Shelly Turner (and daughter Jen)  Gender: F  Age range: Shelly 39-45, Jen is 16 but she is not present.  Ethnicity: any  Case Information  Setting: pediatrician’s office, 4:00 PM  SP opening statement:  My daughter passed out during a lacrosse game today. The school nurse said I should bring her to the pediatrician right away.  History of present illness:  What happened: You were not present but this is what the nurse told you. 16-year-old Jen had just finished playing a game of lacrosse which she claims was her best game ever (she plays goalie). She also ran 2 miles prior to the game in order to warm up. About 40 minutes after finishing the game, she was standing with her teammates waiting for you to pick her up when she fainted.  Where: The game took place on the high school campus.    When: She finished the game at 11:30 am and the incident occurred at 12:10pm.  Symptoms: There was no history of palpitations nor chest pain. She described having blurry vision, feeling weak. Her teammates told her that she passed out for about 30 seconds. They didn’t see any jerking episodes. When she woke up, she was pale, and a little confused, but it went away quickly.  Past medical history:  She has never told you that she has been dizzy before and she has no past medical history of problems like this. Her pediatrician diagnosed her with a heart murmur as a baby (you were not living in the DC area then), but she grew out of it by the time she started elementary school.  Family medical history:  Her grandmother died at 81 years of age of ovarian cancer, paternal grandmother had a stroke at 50 years of age. Her paternal grandfather has hypercholesterolemia (high cholesterol) and her maternal grandfather has diabetes mellitus. There is no history of fainting in the family.  Social history:  Jen is a high school junior. She makes As and Bs in school and is hoping for a college lacrosse scholarship. She is very athletic and exercises when it is not lacrosse season. To your knowledge, she has never tried cigarettes, drugs or alcohol. She lives at home with you, your mother, and her twin brother, John. Your mother is 87 and cannot drive. She has a lot of healthcare issues that require you to take her to different specialist appointments. You have taken the maximum days allowed for ASSLA (sick leave from work) to take care of her. Your boss has threatened to terminate you if you take any more days off.  Diet: For breakfast, she had milk, an orange and toast at 5:00am. For lunch she had a chicken caesar salad.    Physical examination findings:  The learner will have this information: Jen’s physical examination is normal.  Diagnosis:  The learner should tell you it is simple (neurocardiogenic) fainting. No referral to cardiologist  How you feel about the situation:  At first you were surprised. It seemed like the coach was overreacting to a simple instance of fainting. Now that you’ve had several hours to think about it, you are starting to get scared. Could this be a serious health condition that could have serious implications for your daughter’s long term goals?  Most concerning factor:  You just read a Parenting magazine article about sudden death in athletes. A friend of your daughter's was recently taken off of the field via EMS for chest pain; she remains benched while an extensive workup is pursued. You don't know the details but you are very anxious and upset that the same thing could happen to Jen. She did have a heart murmur when she was a baby.  Patient presentation:  Remember that this encounter “begins” in the middle. You and the resident have to imagine that the resident has already taken your history. He/she stepped out to answer a page and now is walking back in the room to counsel you. You have a very worried and anxious affect when the doctor walks in. You don’t cry but your body language and tone of voice should demonstrate that you are concerned.  Based on the resident’s plan, you will have one of two anxious responses. (1) If the resident tells you that nothing is seriously wrong with your daughter, you are not relieved and ask for a cardiology referral. After all, can the resident guarantee he/she is right? Isn’t it better to get a second opinion? (2) If the doctor tells you that Jen could have a serious condition and needs to see a specialist, you are anxious because it could be serious AND because missing another day from work may get you fired.  The point of the training is to enable the resident to better handle a “difficult” encounter. Your anxiety level should ultimately decrease for those residents who employ some of the following communication strategies:  Elicit emotions via direct and indirect questioning   - Direct: How does this make you feel? - Patient explanatory model: “Do you have any thoughts on what’s behind this?   Offer support and empathy.   - Name the affect: (You are sad/mad, etc.) - Validate (Your child had fainted and you are worried something bad will happen; that’s a lot for any parent to take” - Align (I want to do everything in my power to help you feel assured)   Use nonverbal behaviors that convey attentive listening   - Thoughtful nodding - Occasional Silence - Few hand gestures - Adequate facial expression - Leaning forward   If the doctor does not attempt any of these strategies, (1) for no referral, end your push for the specialist consultation with: “I will think it over tonight, but I can’t guarantee that I won’t seek a specialist tomorrow.” (2) If the doctor recommends you see a specialist, reluctantly agree to whatever strategy the resident comes up with for dealing with your work situation.  Additional Materials:  SP Checklist/scoring rubric  **Presenting situation/door chart information**  Patient Name: Shelly Turner and 16-year-old daughter, Jen. This is an established patient of yours. She was diagnosed with a heart murmur as a baby but grew out of it by the time she started elementary school.  Location: Pediatrician’s office  Time/Date: 4:00 pm  Hx: Jen fainted today 40 minutes after a lacrosse game (no SOB, chest pain or palpitation) with reassuring PMH (no history of cardiac or seizures), family history (no SIDS, unexplained death, drowning or car accidents, pacemakers) and normal examination. She has benign vasovagal syncope. Mother is anxious.  Medical Information: Jen’s physical examination is normal  Task: You have already met with the family, obtained the history portion and performed the physical examination. During that time the mother’s emotions displayed were fear and surprise. You had to step outside to answer a page and now you will counsel the family.  Please counsel Jen’s mother, Shelly, as appropriate.  Time: 10 minutes |
| --- |

| **SP Script for pre test Case 2—Jillian and Adam Roper***  Identifying Case Factors:  Presenting complaint: My son fainted during a basketball game today.  Length of patient encounter: 10 minutes  Length and type of learner post-encounter (if applicable): 5 mins SP checklist assessment  Learning objectives/case purpose: Evaluation of a program that teaches residents to assess syncope in adolescents  Diagnosis: Referral to cardiologist  Learner level: Pediatric Residents  Type of Case:  Teaching  Assessment  Communication  History only  SP Demographics:  Name: Jillian Roper (and son Adam)  Gender: F  Age range: Jillian 39-45, Adam is 18 but he is not present.  Ethnicity: any  Case Information  Setting: pediatrician’s office, 3:00 pm  SP opening statement:  My son fainted during a basketball game today. The coach says Adam needs clearance from his doctor before he will allow him to play again.  History of present illness:  What happened: You were not at the game, but this is what the coach told you. Adam was playing basketball and "fainted" when the loud buzzer went off at the end of the first half. He was in the middle of a fast break- running toward the basket and closely guarded by another player. The other player stated there was no contact, and no foul was called when Adam fell to the floor. (Adam plays guard on the team.)  Where: The game took place on the high school campus.    When: He played the game at 9:30 am.  Symptoms: He had no symptoms before he fainted. He woke up after about 20 seconds, according to his team members, unsure of what had happened. When he woke up, he was a little pale and “foggy headed,” but he came back to normal quickly. He did not feel any chest pains, and none of the observers indicated that there were any jerking episodes. He wanted to play, but the coach had him sit out the second half.  Past medical history:  He has never fainted before and he has never had any problems like this. He had sinus arrhythmia noted on a pre-sport evaluation. He had an upper respiratory infection 2 weeks ago. The pediatrician said it was a viral infection and no prescription was needed.  Family medical history:  Adam’s older brother drowned at age 21 while at a Reserve Officers' Training Corps  (ROTC) boot camp, unclear circumstances. His maternal grandfather has high blood pressure and diabetes.  Social history:  Adam is a high school senior. He has a basketball scholarship to Georgetown. He makes good grades. You know he tried beer at a friend’s house about 6 months ago but you do not believe he has tried smoking or drugs. He lives at home with you and his older sister, Vanessa, age 20. You are a single mother who has taken the maximum days allowed for ASSLA (sick leave from work) to take care of Emily who has asthma. Your boss has threatened to terminate you if you take any more days off.  Diet: He has a normal teenager diet. For breakfast, he had orange juice, oatmeal and yogurt at 5:30am. For lunch he had a three slices of pizza and a decaf soda.  Physical examination findings:  The resident will have this information: Adam’s physical examination is normal.  Diagnosis:  The learner should say it is cardiac disease. Referral to cardiologist is needed.  How you feel about the situation:  At first you were surprised. It seemed like the coach was overreacting to a simple instance of fainting. Now that you’ve had several hours to think about it, you are starting to get scared. Could this be a serious health condition that could have serious implications for your son’s long term goals?  Most concerning factor:  You are very worried. It seemed like everything was going so well for Adam. He has a scholarship to Georgetown, which is good because you are a single mom and will have difficulty paying for college without it. Next week is the championship game and the Georgetown coach will be there to watch Adam. You’re afraid that if the high school coach won’t let Adam play under these circumstances, it must be really bad. You’ve already lost one son; you can’t imagine losing another.  Patient presentation:  Remember that this encounter “begins” in the middle. You and the resident have to imagine that the resident has already taken your history. He/she stepped out to answer a page and now is walking back in the room to counsel you. You have a very worried and anxious affect when the doctor walks in. You don’t cry but your body language and tone of voice should demonstrate that you are concerned.  Based on the resident’s plan, you will have one of two anxious responses. (1) If the resident tells you that nothing is seriously wrong with your son, you are not relieved and ask for a cardiology referral. After all, can the resident guarantee he/she is right? Isn’t it better to get a second opinion? (2) If the doctor tells you that Adam could have a serious condition and needs to see a specialist, you are anxious because it could be serious AND because missing another day from work may get you fired.  The point of the training is to enable the resident to better handle a “difficult” encounter. Your anxiety level should ultimately decrease for those residents who employ some of the following communication strategies:  Elicit emotions via direct and indirect questioning   - Direct: How does this make you feel? - Patient explanatory model: “Do you have any thoughts on what’s behind this?   Offer support and empathy.   - Name the affect: (You are sad/mad, etc.) - Validate (Your child had fainted and you are worried something bad will happen; that’s a lot for any parent to take” - Align (I want to do everything in my power to help you feel assured)   Use nonverbal behaviors that convey attentive listening   - Thoughtful nodding - Occasional Silence - Few hand gestures - Adequate facial expression - Leaning forward   If the doctor does not attempt any of these strategies, (1) for no referral, end your push for the specialist consultation with: “I will think it over tonight, but I can’t guarantee that I won’t seek a specialist tomorrow.” (2) If the doctor recommends you see a specialist, reluctantly agree to whatever strategy the resident comes up with for dealing with your work situation.  Additional Materials:  SP Checklist/scoring rubric  **Presenting situation/door chart information**  Patient Name: Jillian Roper and son Adam. This is an established patient of yours who was seen for a reassuring school physical 3 months ago. He was noted to have sinus arrhythmia, normal variation, in the past.  Location: Pediatrician’s office  Time/Date: 3:00 pm, today  Complaint: Adam "fainted" when the loud buzzer went off at the end of the first half of his basketball game, his older brother drowned at age 21 while at a ROTC boot camp, unclear circumstances. These are very concerning and require a cardiology referral.  Medical Information: Adam’s physical examination is normal.  Task: You have already met with the family, obtained the history portion and performed the physical examination. During that time the mother’s emotions displayed were fear and sadness. You had to step outside to answer a page and now you will counsel the family.  Please counsel Adam’s mother, Jillian, as appropriate.  Time: 10 minutes  **SP Script for Intervention Case 2—Laura and Nathan Hopkins***  Identifying Case Factors:  Presenting complaint: My son passed out during baseball practice today.  Length of patient encounter: 10 minutes  Length and type of learner post-encounter (if applicable): 5 mins SP checklist assessment  Learning objectives/case purpose: Evaluation of a program that teaches residents to assess syncope in adolescents  Diagnosis: Referral to cardiologist  Learner level: Pediatric Residents  Type of Case:  Teaching  Assessment  Communication  History only  SP Demographics:  Name: Laura Hopkins (and son Nathan)  Gender: F  Age range: Laura 39-45, Nathan is 17 but he is not present.  Ethnicity: any  Case Information  Setting: pediatrician’s office  SP opening statement:  My son passed out today during baseball practice. The coach said he needs clearance from the doctor before he can come back.  History of present illness:  What happened: You were watching Nathan play baseball when Nathan, who is the third baseman, ran to catch a fly ball and suddenly collapsed. He did not appear to trip and there was no contact with another player.  Where: The game took place on the high school campus.    When: He played the game at 9:30 am. The incident occurred at 11 am.  Symptoms: He described having blurry vision, feeling weak and then waking up 20 seconds later. He did not feel any chest pains or palpitations, and you didn’t observe any jerking episodes. When he woke up, he was a little pale and “disoriented,” but he came back to normal quickly. He wanted to play, but his coach had him sit out the rest of the game.  Past medical history:  Nothing like this has ever happened before. He is a very competitive athlete who has been playing baseball since 7 years of age. Nathan was diagnosed with ADHD when he was 8. He usually takes Adderall therapy (20 mg 1 time per day) but since he didn’t have classes today, he did not take it.  Family medical history:  Nathan’s older brother drowned at age 14 while at summer camp. His maternal grandfather has high blood pressure and diabetes.  Social history:  Nathan is a high school junior. He is hoping for a college scholarship. He is also active in the debate team and is interested in going to law school. He does well in school. You know he tried cigarettes once, but you don’t think he has tried drugs or alcohol. He lives at home with you, your husband, and his younger sister, Lisa, age 12. Your husband injured his back at work and is at home on disability. He needs a lot of assistance, cannot drive, and has to go to PT regularly. You have taken the maximum days allowed for ASSLA (sick leave from work) to take care of your husband. Your boss has threatened to terminate you if you take any more days off.  Diet: Nathan has a healthy diet. For breakfast, he had juice, banana and yogurt with granola at 5:30am. For lunch he had a grilled cheese sandwich and tomato soup.  Physical examination findings:  The learner will have this information: Nathan’s physical examination is normal.  Diagnosis:  The learner should tell you it is a cardiac disease. Referral to cardiologist is needed.  How you feel about the situation:  At first you were surprised. It seemed like the coach was overreacting to a simple instance of fainting. Now that you’ve had several hours to think about it, you are starting to get scared. Could this be a serious health condition that could have serious implications for your son’s long term goals?  Most concerning factor:  Nathan has always been so active. You just can’t imagine how it will affect him if he can’t play baseball. You’re afraid that if the coach won’t let him play under these circumstances, it must be really bad. You’ve already lost one son; you can’t imagine losing another.  Patient presentation:  Remember that this encounter “begins” in the middle. You and the resident have to imagine that the resident has already taken your history. He/she stepped out to answer a page and now is walking back in the room to counsel you. You have a very worried and anxious affect when the doctor walks in. You don’t cry but your body language and tone of voice should demonstrate that you are concerned.  Based on the resident’s plan, you will have one of two anxious responses. (1) If the resident tells you that nothing is seriously wrong with your son, you are not relieved and ask for a cardiology referral. After all, can the resident guarantee he/she is right? Isn’t it better to get a second opinion? (2) If the doctor tells you that Nathan could have a serious condition and needs to see a specialist, you are anxious because it could be serious AND because missing another day from work may get you fired.  The point of the training is to enable the resident to better handle a “difficult” encounter. Your anxiety level should ultimately decrease for those residents who employ some of the following communication strategies:  Elicit emotions via direct and indirect questioning   - Direct: How does this make you feel? - Patient explanatory model: “Do you have any thoughts on what’s behind this?   Offer support and empathy.   - Name the affect: (You are sad/mad, etc.) - Validate (Your child had fainted and you are worried something bad will happen; that’s a lot for any parent to take” - Align (I want to do everything in my power to help you feel assured)   Use nonverbal behaviors that convey attentive listening   - Thoughtful nodding - Occasional Silence - Few hand gestures - Adequate facial expression - Leaning forward   If the doctor does not attempt any of these strategies, (1) for no referral, end your push for the specialist consultation with: “I will think it over tonight, but I can’t guarantee that I won’t seek a specialist tomorrow.” (2) If the doctor recommends you see a specialist, reluctantly agree to whatever strategy the resident comes up with for dealing with your work situation.  Additional Materials:  SP Checklist/scoring rubric  **Presenting situation/door chart information**  Patient Name: Laura Hopkins and son Nathan, 17 years old. This is an established patient of yours. Nathan was diagnosed with ADHD when he was 8. He usually takes Adderall therapy (20 mg 1 time per day) but since he didn’t have classes today, he did not take it.  Location: Pediatrician’s office  Time/Date: 2:00 pm, today  Hx: Adam passed out today during a baseball game. He ran to catch a fly ball and suddenly collapsed, Nathan’s older brother drowned at age 14 while at summer camp. These are very concerning and require a cardiology referral.  Medical Information: Nathan’s physical examination is normal.  Task You have already met with the family, obtained the history portion and performed the physical examination. During that time the mother’s emotions displayed were fear and sadness. You had to step outside to answer a page and now you will counsel the family.  Time: 10 minutes  **SP Script for Post test Case 2—Nancy & John Adams***  Identifying Case Factors:  Presenting complaint: My son passed out during soccer game today.  Length of patient encounter: 10 minutes  Length and type of learner post-encounter (if applicable): 5 mins SP checklist assessment  Learning objectives/case purpose: Evaluation of a program that teaches residents to assess syncope in adolescents  Diagnosis: Referral to cardiologist  Learner level: Pediatric Residents  Type of Case:  Teaching  Assessment  Communication  History only  SP Demographics:  Name: Nancy Adams (and son John)  Gender: F  Age range: Nancy 39-45, John is 17 but he is not present.  Ethnicity: any  Case Information  Setting: pediatrician’s office, 2:00 PM  SP opening statement:  My son passed out today during the soccer game. The coach said the doctor would have to give him clearance before he can play again.  History of present illness:  What happened: You were at work. The coach called you and said that during the soccer game today, John was running towards the goal when he collapsed just before kicking the ball. He did not appear to trip and there was no contact with another player (John plays striker). It is a hot, humid May day. The high temperature today is supposed to be 85 degrees F.  Where: The game took place on the high school campus.    When: He played the game at 9:30 am. The incident occurred at 10 am.  Symptoms: He woke up after about 20 seconds, unsure of what had happened, and he was a little pale. No one observed anything unusual and John didn’t mention any other symptoms. He wanted to play, but his coach had him sit out the rest of the game.  Past medical history:  Nothing like this has ever happened before. He is a very competitive athlete who has been playing soccer since 8 years of age. John was diagnosed with ADHD when he was 5. He usually takes Adderall therapy (20 mg 1 time per day) but since he didn’t have classes today, he did not take it.  Family medical history:  No history of sudden death or syncope. His maternal grandfather has high blood pressure and diabetes.  Social history:  John is a high school junior. He is hoping for a college scholarship. He is also active in the student council. He does well in school and you do not believe he has tried drugs or alcohol. You divorced John’s father when John was 11. John lives at home with you, your mother, and his older sister, Anne, age 19. Your mother is 87 and cannot drive. She has a lot of healthcare issues that require you to take her to different specialist appointments. You have taken the maximum days allowed for ASSLA (sick leave from work) to take care of her. Your boss has threatened to terminate you if you take any more days off.  Diet: He generally eats a well-rounded diet. For breakfast he had milk, hard- boiled egg, and toast at 5:30am. For lunch he had a Chipotle chicken burrito.  Physical examination findings:  The learner will have this information: John’s physical examination is normal.  Diagnosis:  The learner should say it is a cardiac disease. Referral to cardiologist is needed.  How you feel about the situation:  At first you were surprised. It seemed like the coach was overreacting to a simple instance of fainting. Now that you’ve had several hours to think about it, you are starting to get scared. Could this be a serious health condition that could have serious implications for your son’s long term goals?  Most concerning factor:  You are very worried that John’s health and education could be in jeopardy. At next week’s game, you have someone filming John for college demo tapes. Now, you’re afraid he may never play again.  Patient presentation:  Remember that this encounter “begins” in the middle. You and the resident have to imagine that the resident has already taken your history. He/she stepped out to answer a page and now is walking back in the room to counsel you. You have a very worried and anxious affect when the doctor walks in. You don’t cry but your body language and tone of voice should demonstrate that you are concerned.  Based on the resident’s plan, you will have one of two anxious responses. (1) If the resident tells you that nothing is seriously wrong with your son, you are not relieved and ask for a cardiology referral. After all, can the resident guarantee he/she is right? Isn’t it better to get a second opinion? (2) If the doctor tells you that John could have a serious condition and needs to see a specialist, you are anxious because it could be serious AND because missing another day from work may get you fired.  The point of the training is to enable the resident to better handle a “difficult” encounter. Your anxiety level should ultimately decrease for those residents who employ some of the following communication strategies:  Elicit emotions via direct and indirect questioning   - Direct: How does this make you feel? - Patient explanatory model: “Do you have any thoughts on what’s behind this?   Offer support and empathy.   - Name the affect: (You are sad/mad, etc.) - Validate (Your child had fainted and you are worried something bad will happen; that’s a lot for any parent to take” - Align (I want to do everything in my power to help you feel assured)   Use nonverbal behaviors that convey attentive listening   - Thoughtful nodding - Occasional Silence - Few hand gestures - Adequate facial expression - Leaning forward   If the doctor does not attempt any of these strategies, (1) for no referral, end your push for the specialist consultation with: “I will think it over tonight, but I can’t guarantee that I won’t seek a specialist tomorrow.” (2) If the doctor recommends you see a specialist, reluctantly agree to whatever strategy the resident comes up with for dealing with your work situation.  Additional Materials:  SP Checklist/scoring rubric  **Presenting situation/door chart information**  Patient Name: Nancy Adams and son John. This is an established patient of yours who was diagnosed with attention deficit hyperactivity disorder **(**ADHD) when he was 5. He usually takes Adderall therapy (20 mg 1 time per day) but since he didn’t have classes today, he did not take it.  Location: Pediatrician’s office  Time/Date: 2:00 pm, middle of May—the forecast is 85 degrees F for the high temperature  Hx: During the soccer game today, John was running towards the goal when he collapsed just before kicking the ball. No history of sudden death or syncope, but this is still a very concerning event and requires a cardiology referral.  Medical Information: John’s physical examination is normal.  Task You have already met with the family, obtained the history portion and performed the physical examination. During that time the mother’s emotions displayed were fear and sadness. You had to step outside to answer a page and now you will counsel the family.  Time: 10 minutes |
| --- |
